# *ERG* Rearrangement Is Associated with Prostate Cancer-Related Death in Chinese Prostate Cancer Patients

**DOI:** 10.1371/journal.pone.0084959

**Published:** 2014-02-07

**Authors:** Mei Qi, Xiaoqing Yang, Fan Zhang, Tao Lin, Xiubin Sun, Yanjiang Li, Huiqing Yuan, Yubo Ren, Juan Zhang, Xiaomin Qin, Bo Han

**Affiliations:** 1 Department of Pathology, Shandong University Medical School, Jinan, China; 2 Department of Orthodontics, Shandong University School of Stomatology, Jinan, China; 3 Department of Surgery, The Central Hospital of Jinan, Jinan, China; 4 Department of Statistics, Shandong University School of Public Health, Jinan, China; 5 Department of Urology, The Affiliated Hospital of Qingdao University Medical College, Qingdao, China; 6 Department of Biochemistry, Shandong University Medical School, Jinan, China; 7 Department of Pathology, Liaocheng General Hospital, Liaocheng, China; 8 Department of Pathology, Shandong University Qilu hospital, Jinan, China; Northwestern University, United States of America

## Abstract

Recently, ETS-related gene (*ERG*) gene rearrangements, phosphatase tensin homologue (*PTEN*) deletions and *EGFR* family aberrations were characterized as potential biomarkers for prostate cancer (PCa) patient management. Although *ERG* gene rearrangement has been identified in approximately 50% of localized prostate cancers in western countries, the prognostic significance of this critical molecular event remains unknown in Chinese patients. Using fluorescence in situ hybridization (FISH) and immunohistochemistry, we evaluated *ERG*, *PTEN* and *EGFR* family aberrations in a cohort of 224 Chinese prostate cancer patients diagnosed in transurethral resection of the prostate (TUR-P). Overall, *ERG* rearrangement was detected in 23.2% (44/190) cases, of which 54.5% (24/44) showed deletion of the 5′end of *ERG*. *PTEN* deletion was identified in 10.8% (19/176) cases. Amplification of *EGFR* and *HER2* genes was present in 1.1% (2/178) and 5.8% (10/173) of cases, respectively. Significant correlation between *ERG* rearrangement and *PTEN* deletion was identified in this cohort. *EGFR* and *HER2* aberrations occurred more frequently in PCas without *ERG* rearrangement than in those with *ERG* rearrangement, although this did not reach statistical significance. Overall, *ERG* rearrangement was associated with pre-operative PSA values (P = 0.038) and cancer-related death (P = 0.02), but not with the age, clinical T stage, Gleason score, or Ki-67 labeling index (LI). Notably, multivariate analysis including known prognostic markers revealed *ERG* rearrangement was an independent prognostic factor (P = 0.022). Additionally, *ERG* rearrangement status was helpful to identify patients with poor prognosis from PCa group with low Ki-67 LI. In summary, we reported that *ERG* rearrangement was associated with cancer-related death in Chinese PCa patients. Determination of *ERG* rearrangement status allows stratification of PCa patients into different survival categories.

## Introduction

Prostate cancer (PCa) is a heterogeneous disease with a variable natural history [1], [2]. It is estimated that only a small fraction of patients suffers from potential life-threatening disease that requires aggressive treatment. Currently, the established prognostic factors (Gleason score, pathological stage and serum prostate-specific antigen (PSA)) cannot precisely distinguish clinically aggressive PCas from clinically indolent ones [3], [4]. Thus, novel prognostic biomarkers are urgently needed for PCa patient management.

Recently, recurrent gene fusions involving the ETS family of transcription factors, *ERG*,*ETV1*, *ETV4*, *ETV5* and *ELK4*, fused to androgen-regulated gene *TMPRSS2* or other upstream partners, have been identified in the majority of PCas in western countries [5]–[8]. Among these aberrations, *ERG* rearrangement, which mostly results from *TMPRSS2-ERG* fusion, is the most prevalent and occurs in approximately 50% of localized PCas [8]. As *TMPRSS2* and *ERG* are located ∼3 Mb apart on chromosome 21, the rearrangement between them occurs either through insertion or by an interstitial deletion (EDel) [6]. *TMPRSS2-ERG* fusion leads to over-expression of ERG, which may play a critical role in PCa development [8]. To date, the prognostic significance of *ERG* rearrangement in PCa remains controversial. Although several studies have indicated that *ERG* rearrangement confers a worse prognosis [9]–[12], others found either a favorable prognostic association[13]–[17] or no association with clinical outcome [18]–[20]. Of note, most of these data are from Caucasian patients in western countries. Although the emerging data suggested the distinct prevalence of *ERG* rearrangement in PCas among different ethnic groups [21], [22], survival analysis of *ERG* aberrations is rare in Asian populations.

*PTEN* (phosphatase and tensin homolog deleted on chromosome 10) is a key tumor suppressor gene in PCa [23]. Deletion of the *PTEN* occurs in 20–70% of PCas and has been linked to rapid tumor progression and early recurrence [24]. Previously, we and others reported the significant association between *PTEN* deletion and *ERG* rearrangement both in localized and metastatic PCas [25]. Recent clinical data have suggested that *PTEN* deletion and *ERG* rearrangement could be used for prognostic stratification of PCa patients [26].

The epidermal growth factor receptor (*EGFR*) and *HER2* belong to the *EGFR* family and are known to regulate cell proliferation, differentiation, angiogenesis, and survival. Amplification and over-expression of *EGFR* and *HER2* have been described in PCa and associated with cancer progression, poor prognosis or development of androgen independence [27]. Yet so far, the link between *ERG* rearrangement and genetic aberrations of *EGFR* and *HER2* remains unclear.

The Ki-67 LI is a classical proliferation marker and has been found to be a predictor of outcome for PCa patients treated with radical prostatectomy [28], [29] or radiotherapy. Ki-67 has emerged as one of the global predictive markers of treatment outcome in PCa patients.

The aim of the current study was to investigate whether *ERG* rearrangement was associated with a more aggressive phenotype of PCa. Herein, we systematically characterized the frequency and prognostic significance of *ERG* rearrangement in a large cohort of Chinese PCa patients (n>200). We further determined whether the *ERG* rearrangement can be utilized as a prognostic indicator and provide additional value in prognostic analysis. Additionally, the relationship of *ERG* gene rearrangement with other molecular markers, including *PTEN* deletion and genetic aberrations of *EGFR* and *HER2,* was also investigated.

## Materials and Methods

### Patients

A total of 224 PCa patients who underwent tumor resection by transurethral resection of prostate (TUR-P) were included in our study. The tumor samples were obtained from Qilu Hospital of Shandong University (Jinan, China), The Affiliated Hospital of Qingdao University (Qingdao, China) and Liaocheng General Hospital (Liaocheng, China) between 2003 and 2011. All of these patients were hospitalized due to symptoms of lower tract urinary obstruction. Eighty-five PCa patients in the current study had transrectal ultrasound*-*guided prostate biopsy and 63.5% (54/85) cases had peripheral zone cancer that extended into transition zone. None of the patients received preoperative radiation or androgen deprivation therapy. Anti-androgen flutamide therapy was followed after surgery and follow-up data were available for 190 patients, ranging from 3 to 147 months (mean 47 months). Because the number of non-PCa deaths (n = 17) was limited in this cohort, the prostate cancer-related death approached the all-cause mortality. The clinical and pathological characteristics of 190 PCa cases in our cohort are summarized in Table 1. Three tissue microarrays (TMAs) were assembled using a manual tissue arrayer; for each case, two cores (1.0 mm in diameter) were taken from each representative tumor focus and morphology was verified by three pathologists (M.Q., B.H. and X.Y.). A 4 µm section form each TMA was stained with H&E to verify the presence of tumor in PCa cases. Detailed clinical and pathological profile were obtained from medical records and maintained on a secure relational database with TMA data. Informed written consents were obtained from the PCa patients and this study was approved by the Institutional Review Board at the school of medicine of Shandong University and local ethics.

**Table 1:** Clinicopathological demographics of 190 Chinese prostate cancer patients.

Parameters	Count	Percentage (%)
Age(years)		
<60	29	15
60–69	41	22
≥70	120	63
Gleason scores		
<7	26	14
= 7	70	37
>7	94	49
cT		
≤T2	138	73
T3	30	16
T4	22	11
Preoperative PSA levels(ng/ml)		
≤4	22	12
4–10	27	14
10–20	25	13
>20	116	61
Distant metastasis at diagnosis		
No	150	79
Yes	40	21

### Fluorescence *in situ* Hybridization (FISH)

A previously described dual-color interphase break-apart FISH assay was performed to detect *ERG* rearrangement [25]. Bacterial artificial chromosomes (BACs) were obtained from the BACPAC Resource Center (Oakland, CA), and probes RP11-95I21 (5′ to *ERG*) and RP11-476D17 (3′ to *ERG*) were prepared as described [25]. The integrity and correct localization of all probes were verified by hybridization to metaphase spreads of normal peripheral lymphocytes. To detect *PTEN* deletion, the commercially available DNA probes for cytoband 10q23 (Spectrum Orange *PTEN* locus-specific probe) and region 10p11.1–q11.1 (Spectrum Green centromere of chromosome 10 probe) (LSI *PTEN*/CEP 10; Vysis Inc. Des Plaines,IL, USA) for chromosome identification were utilized. The *PTEN* genomic probe spans 368 kb and starts 166 kb from 5′ end of the gene and extends 98 kb past the 3′ end of the gene. Assessment of *EGFR* and *HER2* gene aberrations was performed using the GLP *EGFR*/CSP 7 probe and GLP *HER2*/CSP17, respectively (GP Medical Technologies, Beijing, China).

Interphase FISH was performed as previously described [25], [30]. Slides were examined using an ImagingZ1 microscope (Carl Zeiss, Oberkochen, Germany). FISH signals were scored manually (100× oil immersion) in morphologically intact and non-overlapping nuclei by two pathologists (B.H., and M.Q.), and a minimum of 50 cancer cells from each site were recorded. Cancer sites with very weak or no signals were recorded as insufficiently hybridized. Cases lacking tumor tissue in all two cores were excluded.

**Figure 1:**
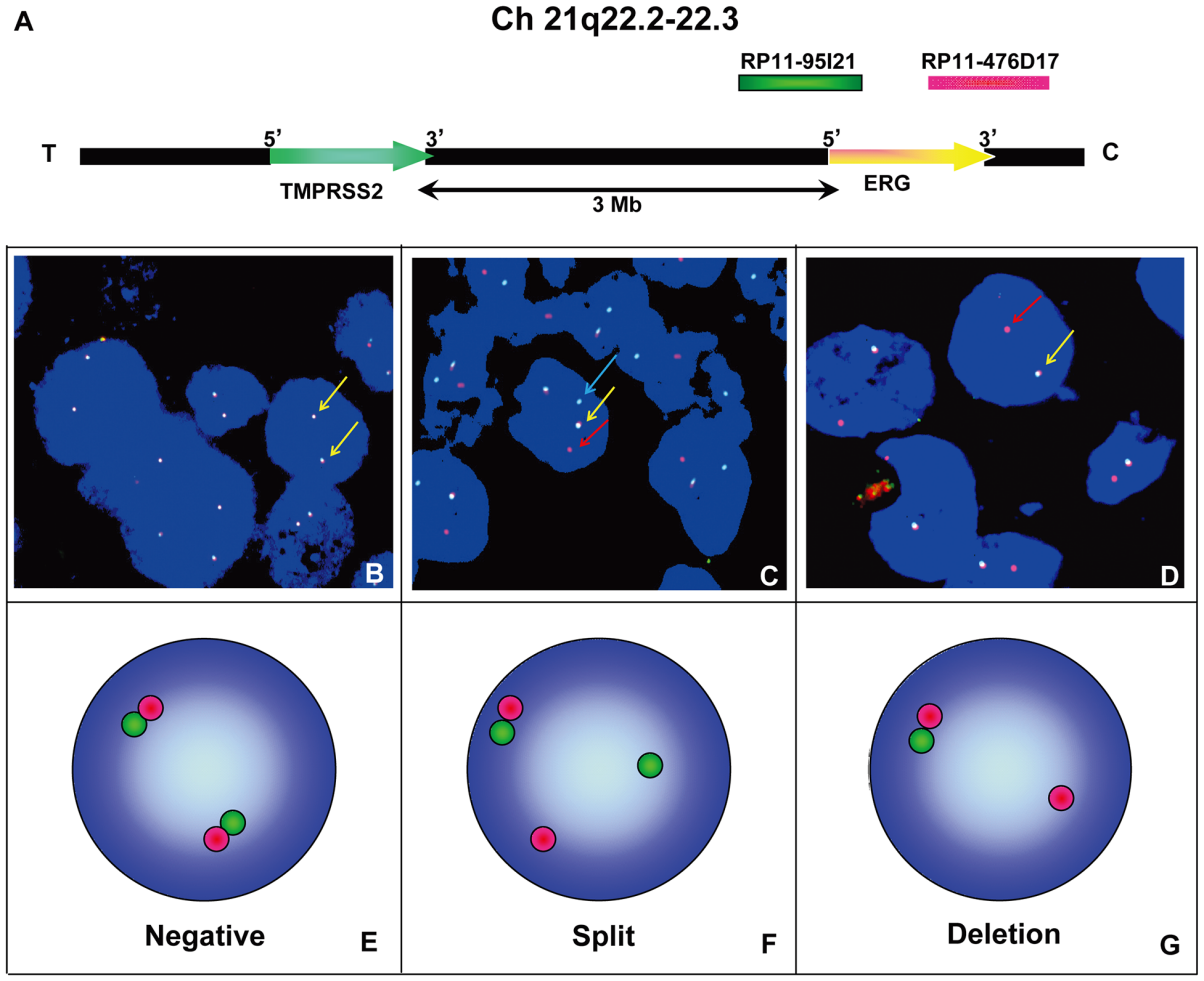
FISH probe design and representative images of *ERG* rearrangement. (A) Schematic map of ‘*TMPRSS2*’ and ‘*ERG*’ position on 21q22.2–22.3. T and C orientate toward the telomeric and centromeric regions, respectively. BACs located 5′ and 3′ to *ERG* were used as probes for interphase FISH. Chromosomal coordinates are from the March 2006 build of the human genome using the UCSC Genome Browser. The *TMPRSS2*and *ERG* loci are separated by approximately 3 Mb. (B) FISH was performed using BACs as indicated with the corresponding fluorescent label on formalin-fixed paraffin-embedded tissue sections for break-apart FISH of the *ERG* gene. (B & E), *ERG* rearrangement negative case, as indicated by two pairs of co-localized green and red signals. (C & F), *ERG* rearrangement positive (translocation) case showed one pair of split 5′ and 3′ signals. (D & G), *ERG* rearrangement positive (with deletion) case showed loss of one green labeled probe 5′ to *ERG*.

**Figure 2:**
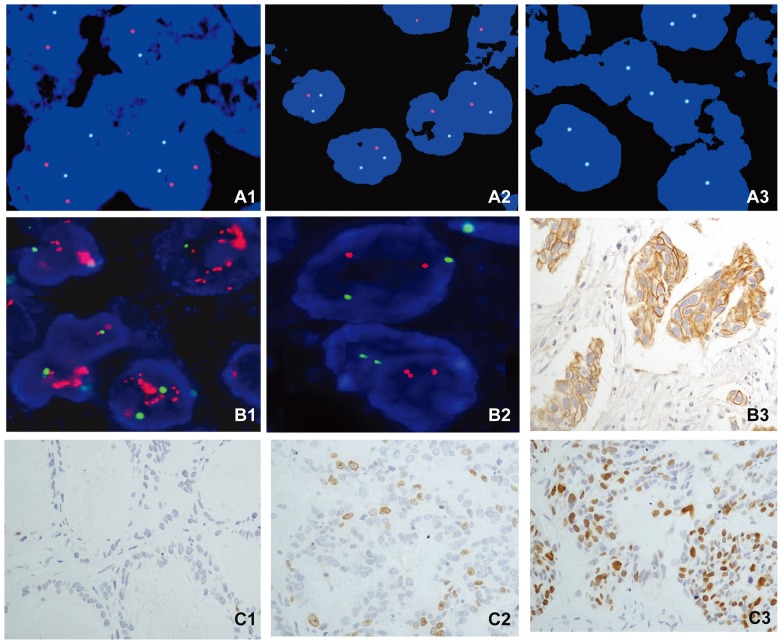
Representative images for IHC staining and FISH analysis of *PTEN,* HER2 and Ki-67 expression in PCa. (A1–A3) FISH images of undeleted, hemizygous and homozygous *PTEN* deletion in PCa. A1, *PTEN* deletion negative case showed both paired red signals (10q23/*PTEN* locus) and green signals in tumor cells. A2, Representative case with *PTEN* hemizygous deletion showed one red signals and pairs of green signals in tumor cells. A3, Representative case with *PTEN* homozygous deletion showed absence of red signals but retained pairs of green signals. For all assays, at least 50 cancer cell nuclei were evaluated. (B1–B3) The detection of HER2 expression by IHC and FISH in PCa. B1, FISH analysis of representative case without *HER2* amplification. B2, FISH analysis of case with *HER2* amplification. B3, HER2 IHC staining shows complete membranous reactivity of strong intensity (3+) in tumor cells (original magnification, ×200). (C1–C3) The Ki-67 staining by IHC in PCa cells. C1, No staining (0) of Ki-67 in tumor cells. C2, Low Ki-67 (LI<10%) nuclear positivity in tumor cells. C3, High Ki-67(LI≥10%) nuclear positivity in tumor cells.

To validate deletion of *PTEN* and amplification of *EGFR* and *HER2*, we utilized a previously documented method with minor modification [25], [31]. Briefly, based on hybridization in five control cores (data not shown), hemizygous deletion of *PTEN* gene was termed as >50% nuclei (mean±3 standard deviations in non-neoplastic controls) containing either one signal of locus probe and ≥2 signals of reference probe (absolute deletion), or two signals of locus probe and ≥4 signals of reference probe (relative deletion). Homozygous deletion of *PTEN* was exhibited by the concurrent lack of the both *PTEN* locus signals and the presence of control signals in >30% of cells. Specimens were considered amplified for *EGFR* when >10% of tumor cells displayed either *EGFR*: CEP 7 ratio >2 or countless tight clusters of signals of the locus probe (3–5copies). *EGFR* copy number gain was defined as a low copy number increase due to chromosome 7 polysomy. Similarly, specimens were considered amplified for *HER2* when >10% of tumor cells displayed either *HER2*: CEP 17 ratio >2 or countless tight clusters of signals of the locus probe (3–5copies). *HER2* copy number gains were defined as a low copy number increase due to chromosome 17 polysomy. Representative FISH images of *ERG* rearrangement were shown in Figure 1. Figure 2A and 2B demonstrated representative cases with *PTEN* deletion as well as *HER2* amplification.

### Immunohistochemistry

Immunohistochemistry (IHC) for PTEN, EGFR and HER2 was performed using a polymer-based method (EnvisionTM +Dual Link System-HRP). Sources and dilutions of primary antibodies were as follows: anti-PTEN (Cell signaling, 1∶100), anti-EGFR (DAKO, 1∶500), anti-HER2 (DAKO, 1∶500) and anti-Ki67 antibody (DAKO, 1∶100). Sections from TMA (4 µm) were deparaffinized and prepared by successive passages through xylene and grade concentration of ethanol as routine procedure, then antigens were retrieved by pressure cooker using a citrate buffer(0.01 M), for 8 minutes 120°C. Endogenous peroxidase activity was blocked by incubation with 0.3% hydrogen peroxide solution for 15 min. The tissue sections were incubated overnight at 4°C with primary antibodies. After a washing in PBS, the sections were treated with EnvisionTM +Dual Link System-HRP reagent at room temperature for 30 min. 3, 3′-Diaminobenzidine tetrahydrochloride was used as the chromogen for 3 minutes and the tissue sections were counterstained with haematoxylin.

The immunostaining of EGFR and HER2 was semiquantitatively evaluated based on intensity of membrane reactivity following the original DAKO Herceptest criteria with a threshold of 10% immunopositive cells. The scoring system was described elsewhere [27]. Evaluation of PTEN was based on the cytoplasmic staining intensity; the tumors were divided into three categories as previously described [25]. Grade 2 showed increased or equal staining intensity compared to the corresponding normal tissue; grade 1 had decreased staining intensity, and grade 0 demonstrated complete absence of staining. The Ki-67 labeling index (LI) was defined as the fraction of tumor cells showing any nuclear Ki-67 immunoreactivity and was considered high if 10% or more of the tumor nuclei were stained. For this purpose, 100–200 tumor cells were analyzed for each case. Representative immunohistochemical images of Ki-67 were shown in Figure 2C.

### Statistical Analysis

Statistical analyses were carried out using the Statistical Package for Social Sciences, version 19.0 (SPSS), with a significance level of 0.05(two-tailed probability). Pearson’s χ2 test and Fisher’s exact test were used to evaluate the associations between *ERG* rearrangement and clinico-pathologic variables as well as other molecular aberrations. Kaplan-Meier analysis was utilized to assess the prognostic value of *ERG* rearrangement in PCa patients. The prognostic value of *ERG* rearrangement was further determined in univariable and multivariable analysis, including PSA values at diagnosis, Gleason score, clinical tumor stage, distant metastasis, Ki-67 LI and *EGFR* family gene aberrations.

## Results

### Frequency of *ERG* Rearrangement, *PTEN* Deletion and *EGFR* Family Aberrations

Overall, *ERG* was rearranged in 23.2% (44/190) of Chinese PCa patients, of which 54.5% (24/44) demonstrated deletion of the 5′end of *ERG*. Interestingly, two out of these 24 cases demonstrated two copies of the 3′-*ERG* signals, suggesting the duplication of *ERG* rearrangement. *PTEN* deletion was identified in 10.8% (19/176) of cases, with hemizygous and homozygous deletions present in 12 of 19 (63.2%) and 7 of 19 (36.8%) cases, respectively. Amplification of *HER2* was identified in 10 of 173 (5.8%) tumors and polysomy of chromosome 17 was noted in 41 of 173 (23.8%) cases. By contrast, only 2 of 178 (1.1%) cases showed amplification of *EGFR* with polysomy of chromosome 7 being present in 18 of 178 (10.1%) tumors.

### Relationships between *ERG* Rearrangement and Clinicopathologic Variables

*ERG* gene rearrangement was significantly associated with preoperative PSA levels in PCa patients (P = 0.038) (Table 2). The incidence of *ERG* rearrangement was significantly lower in patients with Low PSA level (<4 ng/ml) compared with those having medium or high PSA levels. However, no significant correlation was identified between *ERG* rearrangement and age, Gleason score, clinical T stage, or distant metastasis at diagnosis.

**Table 2:** Association of clinicopathologic variables and molecular biomarkers with *ERG* rearrangement.

Variable	*ERG* rearrangement according to FISH (%)		*P*
	Not rearranged(n/%)	Rearranged (n/%)	
All cases	146(76.8)	44(23.2)	
age(years)			
≤65	21(67.8)	10(32.2)	0.173
>65	117(79.0)	31(21.0)	
Pre PSA(ng/ml)			
<4	15(93.8)	1(6.2)	0.038
4–10	11(64.7)	6(35.3)	
>10	103(79.8)	26(20.2)	
Gleason score			
<7	17(73.9)	6(26.1)	0.430
7	51(76.1)	16(23.9)	
>7	78(83.0)	16(17.0)	
Clinical tumor stage			
≤cT2	101(78.3)	28(21.7)	0.607
≥cT3	29(74.4)	10(25.6)	
Metastasis			
No	97(80.8)	23(19.1)	0.585
Yes	37(77.1)	11(22.9)	
Ki-67			
<10%	127(80.5)	31(19.5)	0.385
≥10%	19(73.1)	7(26.9)	
*PTEN* deletion			
Not deleted	130(80.3)	27(19.7)	0.0008
Deleted	7(36.8)	12(63.2)	
*EGFR* amplification			
Not amplified	139(79.0)	37(21.0)	0.883
Amplified	2(100.0)	0(0.0)	
EGFR IHC			
0 and 1+	120(82.3)	25(17.7)	0.779
2+ and 3+	25(80.6)	6(19.7)	
*HER2* amplification			
Not amplified	129(79.1)	34(20.9)	0.671
Amplified	9(90.0)	1(10.0)	
HER2 IHC			
0 and 1+	131(76.6)	40(23.4)	0.541
2+ and 3+	11(100.0)	0(0.0)	

Values not available for all 190 cases.

### Association of *ERG* Rearrangement with Other Molecular Markers

As deletion of *PTEN* and amplifications of *EGFR* and *HER2* are relevant genomic aberrations in PCa, we next explored the association of *ERG* rearrangement with these molecular events in our cohort. As shown in Table 2, the *ERG* rearrangement was present in approximately 63.2% (12/19) of PCa patients with *PTEN* deletion (hemizygous or homozygous). Likewise, *PTEN* deletion occurred more frequently in cases that harbored *ERG* rearrangement (30.8%, 12/39) as compared with those *ERG* rearrangement negative cases (5.1%, 7/137). Overall, a significant association between *PTEN* deletion and *ERG* rearrangement was observed in Chinese PCa cohort (P = 0.0008). Of note, 46/182 (25.2%) PCa cases revealed decreased PTEN protein expression by immunohistochemistry. Concordance between *PTEN* deletion status and PTEN protein expression was also identified in our cohort (data not shown).

Amplification of *EGFR* was identified only in two PCa cases, both of which were negative for *ERG* rearrangement. Similarly, 9 out of 10 (90.0%) PCa cases with *HER2* amplification were absent for *ERG* rearrangement. *ERG* rearrangement was more often present in PCa cases without *HER2* amplification (34/163, 20.9%) than in *HER2*-amplified tumors (1/10, 10.0%) (P = 0.149).

Immunohistochemical overexpressions of EGFR and HER2 were identified in 17.6% (31/176) and 6.0% (11/181) of cases, respectively. HER2 protein overexpression was significantly correlated to amplification of *HER2* (P<0.01). However, there was no correlation between EGFR protein expression and gene amplification (data not shown). *ERG* rearrangement was neither associated with EGFR nor HER2 protein expression.

### Survival Analysis of *ERG* Rearrangement in Relation to Cancer-related Death

**Figure 3:**
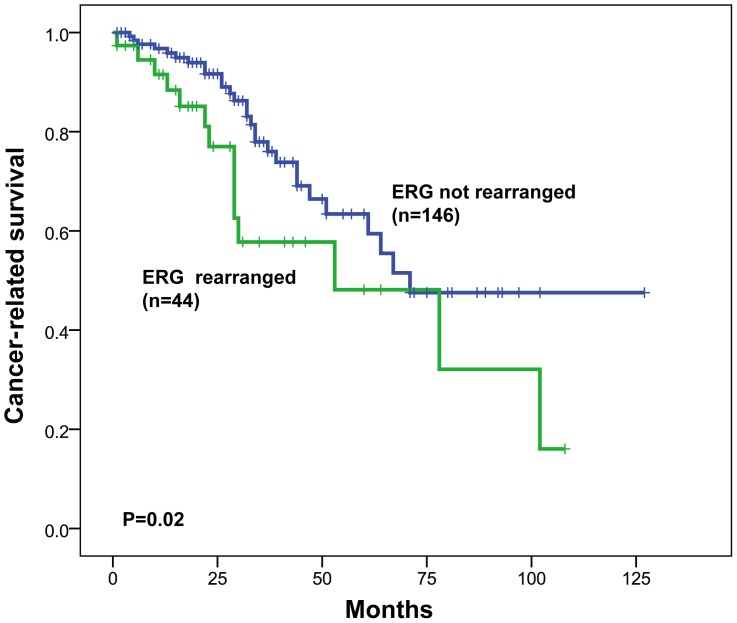
Kaplan-Meier survival curves for PCa patients with and without *ERG* rearrangement. The cancer-related survival rates were compared between patients with and without *ERG* rearrangement using the log-rank test.

To determine whether the presence of *ERG* rearrangement was a prognostic factor for PCa, we compared cancer-related death rates between patients with or without *ERG* rearrangement. On the basis of the Kaplan-Meier survival estimates, the group of patients with *ERG* rearrangement had a much greater rate of mortality than patients who lacked the gene rearrangement (P = 0.02) (Figure 3).

*ERG* rearrangement status was shown to be a significant prognostic predictor of prostate cancer-related survival [HR (95% CI): 3.368 (1.261–8.955), P = 0.015] in univariate analysis (Table 3). PSA values at diagnosis (P = 0.009), Gleason score (P<0.001), clinical tumor stage (P = 0.011), distant metastasis (P = 0.006), Ki-67 LI (P = 0.002), *EGFR* amplification (P = 0.023), and *HER2* amplification (P = 0.001) were also significantly related to cancer-related survival in univariate analysis. Notably, in a multivariate analysis that included known prognostic markers, *ERG* rearrangement status remained a significant predictor (P = 0.022) with a hazard ratio of 2.099 (95% CI: 1.112–3.962) (Table 3).

**Table 3:** Univariate and multivariate analysis of variables associated with survival in PCa patients.

Parameter	Univariate analysis		Multivariate analysis	
	HR(95%CI)	*P*	HR(95%CI)	*P*
age(years)^a^	0.588(0.328–1.053)	0.074	–	–
Pre-PSA	0.601(0.410–0.880)	0.009	Nonsignificancance	
Gleason score	2.297(1.455–3.625)	<0.001	4.680(2.020–10.483)	<0.001
Clincial tumor stage	2.011(1.177–3.435)	0.011	Nonsignificancance	
Metastasis	2.106(1.240–3.577)	0.006	2.897(1.236–6.789)	0.014
Ki-67	2.592(1.435–4.682)	0.002	2.641(1.084–6.435)	0.019
*HER2*amplification	6.687(2.253–19.844)	0.001	Nonsignificancance	
HER2 IHC	3.240(0.998–10.527)	0.05	Nonsignificancance	
*EGFR*amplification	5.255(1.259–21.929)	0.023	Nonsignificancance	
*ERG* rearrangement	3.368(1.261–8.955)	0.015	2.099(1.112–3.962)	0.022

HR = hazard ratio; CI = confidence interval; PSA = prostate-specific antigen.

a not included in multivariate analysis.

### Prognostic Relevance of *ERG* Rearrangement and Ki-67 LI

**Figure 4:**
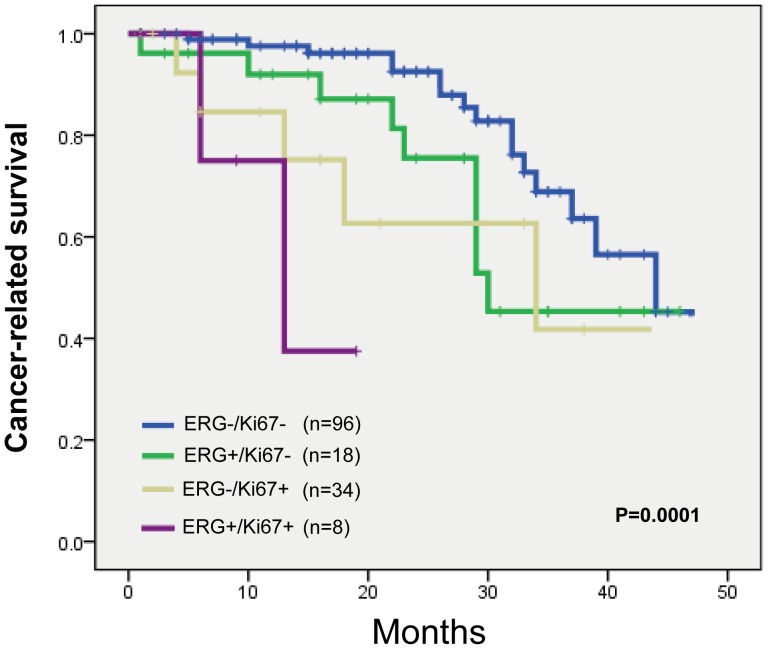
Kaplan-Meier curves illustrating cancer related survival among PCa patients. The patients were stratified by *ERG* rearrangement and Ki-67 LI in combination and log-rank test was performed.

We next determined whether combining markers further improved prognostic value. Since Ki-67 is a known strong prognosticator in PCa and has independent predictive value for cancer-related survival in our cohort, we directly compared the prognostic effects of *ERG* rearrangement and Ki-67 LI in combination. For this analysis, we grouped all cancers according to their *ERG* status (not rearranged vs. rearranged) and the Ki-67 Label index status (LI <10% vs LI>10%). Cox regression analyses were therefore conducted using the group with low Ki-67 LI and no *ERG* aberration as the reference. As shown in Figure 4, the largest group, which comprised those who had no *ERG* rearrangement and low Ki-67 LI, had a greater cancer-related survival when compared with the three other groups. Notably, the subset of patients with *ERG* rearrangement and high Ki-67 LI had the worst cancer-related survival.

**Figure 5:**
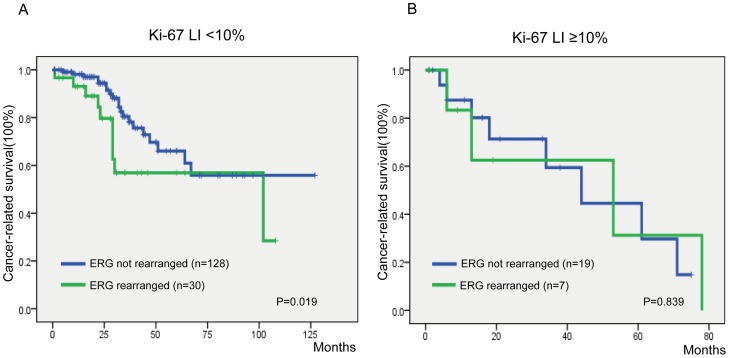
Kaplan-Meier survival analysis of PCa patients in relation to *ERG* rearrangement status. (A) low ki-67 LI (<10%) subgroup, (B) high ki-67 LI (≥10%) subgroup.

We further determined whether *ERG* rearrangement status could be utilized in improving risk stratification of PCa patients with low Ki-67 LI. Kaplan-Meier analysis showed that *ERG* rearrangement status was a prognostic factor in the group of patients with low Ki-67 LI (P = 0.019) (Figure 5A). The median survival of PCa patients with and without *ERG* rearrangement was 69 and 89 months, respectively. However, *ERG* rearrangement status lost its predictive value of outcome in those with high Ki-67 LI (Figure 5B). By contrast, *ERG* rearrangement status was not helpful in identifying high-risk PCa patients with low Gleason score (data not shown).

## Discussion

This is one of the largest series of PCa patients (n>200) reported so far in China analyzing *ERG* rearrangement. Our cohort comprises men treated with TUR-P and all of the study patients had symptoms of lower tract urinary obstruction, therefore representing a select subgroup of clinically recognized PCas. The patients with incidental PCas were excluded from our study. Although more and more PSA-screed PCa patients have been identified in western countries, there are limited data regarding the clinical phenotype or natural history of PCa. Of note, our cohort included a subset of patients with high grade PCas. This differed from most Western patients who were found to have PCa due to PSA screening and were often treated with radical prostatectomy.

Overall, the frequency of *ERG* rearrangement was 23.2% in our cohort and this was comparable with that previously reported by Mao et al [21] and Ren et al [32] in Chinese PCa patients. In consistent with these findings, Kimura et al [17] and Lee et al [33] reported the prevalence of *TMPRSS2-ERG* gene fusion was 16.3% (15/92) in Japanese and 20.9% (53/254) in Korean PCa patients, respectively. Previously, Mosquera et al [34] detected *TMPRSS2-ERG* fusion in 100 Caucasian and non-Caucasian PCa patients undergoing prostate biopsy. They reported that the incidence was significantly different in Caucasians (44/85, 52%) and in non-Caucasians (2/15, 13%). Most recently, using a multicolor FISH assay, Magi-Galluzzi et al [35] found that *TMPRSS2*-*ERG* gene fusion was present in 50% (21/42) of Caucasians, 31.3% (20/64) of African-Americans and 15.9% (7/44) of Japanese (P = 0.003). Collectively, these studies highlighted the low prevalence of *TMPRSS2-ERG* gene fusions in PCa patients in Asia compared with western countries and this disparity at least partially resulted from different genetic background rather than the effects of lifestyle or diet. Of note, the difference may also reflect previous findings that the fusion is less common in transition zone tumors (from which most tumors found in TUR-P samples) than in peripheral zone tumors [17], [36], [37]. Additionally, cohort design and consideration of multifocality might have impacts on the *ERG* rearrangement frequency [38].

So far, the prognostic significance of *ERG* rearrangement in PCa remains contradictory. A series of retrospective studies that sought an association between *TMPRSS2-ERG* and outcome following PSA screened radical prostatectomy gave mixed results. Several published studies have shown that PCa patients with the *TMPRSS2-ERG* gene fusion conferred a higher risk of recurrence, whereas others reported a significant association with a favorable prognosis or a null relationship with clinical outcome. Among patients managed with watchful waiting, *TMPRSS2-ERG* seemed to be associated with worse outcomes. In a meta-analysis including 227 men diagnosed with TUR-P, men with fusion-positive tumors were 1.37 (95% CI, 0.53–3.51) times as likely to experience distant metastases or die from PCa as those negative for the fusion [18]. Discrepancies in the reported prognostic significance of *ERG* rearrangements can be due to cohort design (multifocality and zonal origin of the tumor), fusion detection technique, and are also liable to the primary end point of the study (i.e., biochemical recurrence, overall survival). Therefore, further standardized studies are needed to address this issue. In the current study, we found that *ERG* rearrangement was significantly associated with prostate cancer-related death in Chinese PCa patients. More importantly, *ERG* rearrangement was suggested to be an independent predictor of overall survival in multivariate analyses. It is notable that biochemical recurrence is an imprecise predictor of prostate cancer death. Although PSA might serve as a surrogate endpoint for overall survival, the majority of men with PSA biochemical failure will die of other causes. Ward et al found that in a population of 3897 radical prostatectomy patients, only 8.3% of the men with PSA biochemical failure died of PCa [39]. Therefore, prostate cancer-related death as the primary end point might be more reliable for prognostic analysis. In total, our data supported the concept that if left untreated or lack of initial therapy, *TMPRSS2-ERG* PCa will run a more aggressive clinical course than fusion-negative cancer.

To date, Ki-67 has been widely utilized as a prognostic biomarker in malignancy including PCa. Its independent predictive value for PCa related survival has been confirmed in our study. In line with Antonarakis’s report [40], no significant association between *ERG* rearrangement and Ki-67 LI status was identified. One explanation is that *ERG* rearrangement has different effects on proliferation and invasion *in vitro*, respectively, Ki-67 is a well-known proliferation marker [41]–[43]. By contrast, in 2008, Tomlins et al reported that alternation of *ERG* gene expression significantly affect invasion *in vitro* but has no effect on cellular proliferation [41]. However, when stratifying for Ki-67 status, *ERG* rearrangement was a prognostic factor for cancer- related survival only in PCa patients with low Ki-67 LI. A major clinical challenge in PCa management is the inability to readily distinguish indolent from aggressive tumors in patients who present with low Gleason grade, low tumor volume or low Ki-67 LI. Our data suggested that determination of *ERG* rearrangement status could be helpful in stratification of PCa patients with low Ki-67 LI into different survival categories.

Although gene fusion is a key molecular event in PCa development and *TMPRSS2-ERG* fusion may induce high grade prostatic neoplasia (HGPIN), it is not sufficient to generate a fully transformed phenotype *in vitro* and *in vivo* [41], [44]. Several independent groups have suggested *ERG* may cooperate with other genetic aberrations to promote PCa development and progression, such as *PTEN* haploinsufficiency, enhanced androgen receptor (AR) signaling, overexpression of SOX9 and aberrant phosphoinositide 3-kinase (PI3K) pathway [45]–[47]. Most recently, *TMPRSS2-ERG* was shown to mediate Epithelial to Mesenchymal Transition (EMT) through the induction of WNT signaling pathway via FZD4 as well as ZEB1/ZEB2 axis [48], [49]. Previously, we and others have suggested the significant association between *PTEN* deletion and *ERG* rearrangement both in localized and metastatic PCas in western countries. In this study, we confirmed significant association between *PTEN* deletion and *ERG* rearrangement in Chinese PCa cohort (P = 0.0008). Thus our data highlighted a possible cooperative role of both *ERG* and *PTEN* aberrations in a subset of Chinese PCa cases.

Genetic aberrations of *HER2* and *EGFR* were associated with advanced-stages disease, metastasis and shorten survival in PCa progression. Previous studies have shown the rarity of *EGFR/HER2* amplifications in PCa. Schlomm et al [50] reported that amplification of *EGFR* was present only in 6 of 2,446 PCa cases (0.25%). Similarly, Baek et al [51] found no amplification of the *EGFR* or *HER2* genes in 66 PCa specimens. In our cohort, amplification of *HER2* was present in 5.8% of Chinese PCa cases. Although not reaching statistic significance, *ERG* rearrangement seemed to be more often present in PCa cases without *HER2* amplification than in *HER2*-amplified tumors. Therefore, *HER2* genetic aberration might play a role in a subset of Chinese PCa patients without *ERG* rearrangement.

It should be noted that a small proportion of tumors showing *ERG* arrangement may harbor a fusion between *ERG* and genes other than *TMPRSS2*, including *SLC45A3* or *NDRG1*. On the other hand, it has been suggested that cancers harboring gene fusions occurring by deletion have worse prognosis than those occurring by translocation. However, we did not find significant associations between *ERG* rearrangement by translocation or positive by deletion cancers and outcomes in Chinese PCa patients.

In total, for the first time, we reported that *ERG* rearrangement was associated with cancer-related death in Chinese PCa patients. Determination of *ERG* rearrangement status allows stratification of PCa patients into different survival categories.
